# Sanhua Tea Alleviates Obesity Symptoms by Improving Blood Lipid Levels, Gut Microbiota, and Liver Metabolome in Mice

**DOI:** 10.3390/foods15162912

**Published:** 2026-08-20

**Authors:** Jing Fu, Xinrui Zhao, Jia Liu, Yusen Yang, Heyuan Jiang

**Affiliations:** 1 College of Biological Science and Engineering, Shaanxi University of Technology, Hanzhong 723001, China; 15399100625@163.com (X.Z.); 15891287216@163.com (J.L.); 18609630715@163.com (Y.Y.); 2 Qinba State Key Laboratory of Biological Resources and Ecological Environment (Incubation), Shaanxi University of Technology, Hanzhong 723001, China; 3 Institute of Urban Agriculture, Chinese Academy of Agricultural Sciences, Chengdu 610213, China; jiangheyuan@caas.cn

**Keywords:** Sanhua tea, blood lipid level, gut microbiota, metabolome, obesity

## Abstract

Obesity-related complications have long been significant risk factors threatening human health. Sanhua tea is an instant tea composed of *Rosa rugosa* Thunb., *Jasminum sambac* (L.) Ait., *Citrus aurantium* L. var. *amara* Engl., *Nelumbo nucifera* Gaertn., and *Ligusticum chuanxiong* Hort. This study aimed to investigate the protective effects and underlying mechanisms of Sanhua tea in obese mice. An obesity model was established by feeding C57BL/6J mice a high-fat diet. Mice received intragastric Sanhua tea at doses (500, 1500, and 2500 mg/kg/day) for three weeks. Biochemical analyses indicated that Sanhua tea significantly reduced total cholesterol, triglyceride, low-density lipoprotein cholesterol, and pro-inflammatory cytokine levels in serum. Sanhua tea also alleviated liver oxidative stress by reducing malondialdehyde levels and regulating the activities of related enzymes. Additionally, Sanhua tea maintained gut microbiota diversity and reshaped microbial composition, reducing the Firmicutes/Bacteroidota ratio, increasing beneficial bacterial communities, and significantly decreasing harmful bacterial communities. Metabolomics analysis revealed that Sanhua tea altered liver metabolites, which were significantly associated with specific gut microbes. These metabolites influenced pathways such as ABC transporters, general metabolic pathways, and central carbon metabolism in cancer, contributing to obesity alleviation. In conclusion, Sanhua tea alleviated obesity symptoms in mice by modulating blood lipid levels, liver metabolism, and gut microbiota.

## 1. Introduction

Obesity is characterized not only by excessive fat accumulation but also by increased risk for cardiometabolic diseases, osteoarthritis, dementia, depression, and certain cancers [1], resulting in physiological, pathological, and psychological changes. Since 1990, the number of obese adults worldwide has more than doubled [2], and it is estimated that by 2050, overweight and obese adults will total 3.8 billion, exceeding half of the global adult population [3]. Overweight and obesity are increasingly prevalent among children and adolescents [4]. In recent years, oral medications for obesity have received regulatory approval in several regions. Oral semaglutide, a glucagon-like peptide-1 (GLP-1) receptor agonist, has long been approved for the treatment of type 2 diabetes and has also been evaluated at higher doses for weight management. Despite its therapeutic benefits, semaglutide is associated with gastrointestinal adverse effects, most notably transient nausea and vomiting, which may compromise treatment tolerability and, in some cases, pose additional health risks [5]. Hence, dietary interventions aimed at preventing or mitigating obesity deserve greater attention.

Given the significant side effects associated with pharmaceutical treatments, safer and healthier methods for lowering blood lipid levels are highly desirable [6]. Studies have indicated that tea consumption exerts anti-inflammatory, lipid-lowering, and hypoglycemic effects [7,8], that Chinese herbal teas are beneficial to human health [9], and that flower teas exhibit notable bioactivities [10]. In 1981, “Sanhua Weight Loss Tea,” formulated from *Rosa rugosa* Thunb., *Jasminum sambac* (L.) Ait., *Citrus aurantium* L. var. *amara* Engl., *Nelumbo nucifera* Gaertn., and *Ligusticum chuanxiong* Hort., was developed in Shanghai, China, demonstrating a 70% efficacy rate in promoting weight loss among obese patients [11]. Recently, we have developed instant Sanhua tea based on this formula. In vitro experiments revealed that Sanhua tea inhibited cholesterol esterase and pancreatic lipase activities and bound bile salts, displaying promising lipid-lowering potential [12]. Although the lipid-lowering efficacy of Sanhua tea has been confirmed, its mechanism of action in vivo remains unclear, necessitating further investigation.

Obesity can lead to abnormal lipid levels in the body [13,14] and is directly associated with liver dysfunction. Changes in lipid metabolism represent a critical intermediate pathway connecting obesity to liver health [15], and also increase the risk of cardiovascular diseases [16]. Currently, the composition of gut microbiota and their metabolites is recognized as a key factor influencing the onset and progression of obesity and related diseases [17]. Previous studies have demonstrated that tea consumption regulates gut microbiota [18], alleviating microbiota imbalance induced by a high-fat diet [19]. Specific gut microorganisms and their metabolic products can mitigate obesity through immune modulation [20]. However, no study has yet investigated the effects of Sanhua tea consumption on lipid levels, gut microbiota, and liver metabolites in obese patients.

To further explore the physiological role of Sanhua tea in vivo, we established an obesity model using C57BL/6J mice and corresponding administration groups. By systematically evaluating health indicators, gut microbiota composition, and liver metabolite profiles in obese mice treated with different doses of Sanhua tea, we aimed to comprehensively elucidate the effects of Sanhua tea on physiological characteristics and obesity alleviation. Additionally, this study sought to investigate the molecular mechanisms underlying these effects and to provide an effective assessment method for the anti-obesity and lipid-lowering efficacy of similar products.

## 2. Materials and Methods

### 2.1. Preparation of Sanhua Tea

*Rosa rugosa* Thunb. was purchased from Bozhou Zicao Tang Tea Industry Co., Ltd. (Bozhou, China). *Jasminum sambac* (L.) Ait. was obtained from Anhui Yaozhiyuan Chinese Herbal Decoction Pieces Co., Ltd. (Bozhou, China). *Citrus aurantium* L. var. *amara* Engl. was sourced from Xi Trading House, Yuzhou District, Yulin City (Yulin, China). *Nelumbo nucifera* Gaertn. and *Ligusticum chuanxiong* Hort. were acquired from Shaanxi Guangtaihe Pharmaceutical Co., Ltd. (Hanzhong, China). The five raw materials were separately pulverized, and 8 g of each was combined in equal proportions. Purified water (1200 mL) was then added at a solid-to-liquid ratio of 1:30 (g/mL), and the mixture was thoroughly homogenized. Extraction was performed in a water bath at 90 °C for 15 min, followed by filtration through four layers of gauze. The remaining residue was subjected to a second extraction under identical conditions. The two resulting filtrates (tea infusions) were pooled, pre-frozen at −30 °C, and subsequently freeze-dried to yield Sanhua tea powder. The resulting powder was stored at 4 °C until further use.

### 2.2. UPLC-ESI-Q TRAP-MS/MS Analysis

UPLC-ESI-Q TRAP-MS/MS analysis was conducted according to the following method [21]. Biological samples were lyophilized using a Scientz-100F lyophilizer (Ningbo Scientz Biotechnology Co., Ltd., Ningbo, China), ground into powder using a Retsch MM 400 grinder (Retsch GmbH, Haan, Germany) at 30 Hz for 1.5 min, and weighed (30 mg) using an MS105DM balance (Mettler-Toledo Instruments Co., Ltd., Zurich, Switzerland). Subsequently, 1500 μL of pre-cooled (−20 °C) 70% methanolic internal standard extract was added, vortexed for 30 s every 30 min (six times), and centrifuged at 12,000 rpm for 3 min. The supernatant was filtered through a 0.22 μm membrane prior to UPLC-ESI-Q TRAP-MS/MS analysis. The analysis employed an Agilent SB-C18 column (1.8 μm, 2.1 mm × 100 mm, Agilent Technologies, Inc., Santa Clara, CA, USA) with mobile phases of 0.1% formic acid in water (A) and acetonitrile (B). The gradient program was as follows: 95–5% A to B within 9 min, held for 1 min, returned to 95–5% A in 1.1 min, and held for another 2.9 min. The flow rate was set at 0.35 mL/min, the column temperature at 40 °C, and the injection volume at 2 μL. ESI parameters included ion source voltages of 5500 V (positive) and −4500 V (negative), source temperature of 500 °C, GS I at 50 psi, GS II at 60 psi, CUR at 25 psi, and collision-activated dissociation (CAD) set to high. Multiple reaction monitoring (MRM) transitions were monitored using nitrogen as collision gas, and declustering potentials (DPs) and collision energies (CEs) were optimized individually for each metabolite. The extraction, detection, and quantitative analysis of metabolites in the samples were performed by Wuhan Metware Biotechnology Co., Ltd. (Wuhan, China, www.metware.cn).

### 2.3. Establishment of Mouse Obesity Model

Male C57BL/6J mice (6 weeks old) were purchased from Beijing Spefu Biotechnology Co., Ltd. (SCXK (Jing) 2019-0010, Beijing, China) and adaptively fed for 5 days under specific-pathogen-free (SPF) conditions at Kangtai Medical Laboratory Services Hebei Co., Ltd. (Langfang, China). The mice were randomly divided into six groups (*n* = 10 per group): blank control (CON), model control (MC), model + atorvastatin (ATV), model + low-dose Sanhua tea (L-SHT), model + medium-dose Sanhua tea (M-SHT), and model + high-dose Sanhua tea (H-SHT). From week one, the CON group was fed a normal diet, while the other five groups were fed a high-fat diet. At the end of the sixth week, orbital blood samples were collected from all mice to measure total cholesterol (TC), triglycerides (TGs), and low-density lipoprotein cholesterol (LDL-C). Significantly elevated levels of these indicators in the five high-fat diet groups compared to the CON group confirmed successful modeling (Figure S1, *p* < 0.01) [22]. All experimental diets were provided by Kangtai Medical Laboratory Services Hebei Co., Ltd. Animal experiments were approved by the Institutional Animal Care and Use Committee (IACUC) of Kangtai Medical Laboratory Services Hebei Co., Ltd. (approval number: MDL2024-03-08-01).

### 2.4. Experimental Design and Sample Collection

Following successful modeling, each group continued with their respective diets until experiment completion. Food and water intake were monitored daily. The treatment groups received atorvastatin (20 mg/kg/day) or low, medium, and high doses of Sanhua tea (500, 1500, 2500 mg/kg/day, respectively) [22]. The approximate human-equivalent doses corresponding to these doses (40.5, 121.6, and 202.7 mg/kg/day) are not intended as recommended doses for human consumption. Treatments were administered by gavage once daily at a dose of 0.1 mL/10 g body weight for 21 consecutive days. Mouse body weights were recorded every 3 days [23], and weight gain rates were calculated. Atorvastatin was purchased from Shanghai Aladdin Biochemical Technology Co., Ltd. (Shanghai, China). After 21 days, orbital blood samples were collected from all mice. Subsequently, mice were euthanized, and liver, subcutaneous adipose tissue, perirenal adipose tissue, epididymal adipose tissue, and brown adipose tissue were harvested. The weights of each tissue were recorded, and adipose indices were calculated.

### 2.5. Biochemical Analysis

Collected whole blood was allowed to stand at 4 °C for 1 h and then centrifuged at 3800 rpm and 4 °C for 10 min. Serum was separated for analysis of TC, TG, LDL-C, high-density lipoprotein cholesterol (HDL-C), alanine aminotransferase (ALT), and aspartate aminotransferase (AST) [24]. Tumor necrosis factor-α (TNF-α), interleukin-1β (IL-1β), interleukin-6 (IL-6), interleukin-10 (IL-10), free fatty acid (FFA), and pancreatic lipase (PL) levels in serum were measured using ELISA kits. TC, TG, malondialdehyde (MDA), superoxide dismutase (SOD), glutathione peroxidase (GSH-PX), catalase (CAT), fatty acid synthase (FAS), acetyl-CoA carboxylase-1 (ACC-1), lipoprotein lipase (LPL), hepatic lipase (HL), and carnitine palmitoyltransferase-1 (CPT-1) levels in liver tissue were also assessed via ELISA. All reagent kits were provided by Kangtai Medical Laboratory Services Hebei Co., Ltd. (Langfang, China).

### 2.6. 16S rRNA Amplicon Sequencing

Mouse fecal samples (*n* = 3) were collected, and high-throughput sequencing of PCR amplification products was performed by Kangtai Medical Laboratory Services Hebei Co., Ltd. Genomic DNA was extracted from feces and evaluated by 1% agarose gel electrophoresis. Specific barcode-tagged primers 338F (ACTCCTACGGGAGGCAGCAG) and 806R (GGACTACHVGGGTWTCTAAT) were synthesized to amplify the targeted region of the 16S rRNA gene using an ABI GeneAmp^®^ 9700 PCR instrument (Applied Biosystems, Foster City, CA, USA) and TransStart^®^ Fastpfu DNA polymerase (TransGen, AP221-02, TransGen Biotech Co., Ltd., Beijing, China). Amplified products were fragmented, PCR-amplified again to enrich library templates, and used to construct Illumina PE250 sequencing libraries. The template DNA fragments were subsequently sequenced on an Illumina PE250 platform. Microbial diversity in fecal samples was analyzed by α- and β-diversity indices. α-diversity was evaluated using Ace, Chao1, Shannon, and Simpson indices, whereas β-diversity was assessed by principal coordinate analysis (PCoA) based on UniFrac distances. Stacked plots and histograms depicting relative abundance at phylum and family classification levels were generated to illustrate gut microbiota distribution. Linear discriminant analysis effect size (LEfSe) was employed to identify biomarkers exhibiting significant differences among groups.

### 2.7. Hematoxylin–Eosin (H&E) Staining

Mouse liver tissues (*n* = 3) were collected and rapidly fixed in 4% paraformaldehyde (PFA) for 24 h, dehydrated with graded ethanol, cleared with xylene (Sinopharm Chemical Reagent Co., Ltd., Shanghai, China), and embedded in paraffin. Embedded tissues were sectioned and stained using hematoxylin (MDL, MD911477) and eosin (MDL, MD911467) [25]. Histopathological changes were observed under a microscope (Leica, DM3000, Leica Microsystems CMS GmbH, Wetzlar, Germany).

### 2.8. Quantitative Real-Time Polymerase Chain Reaction (PCR)

Total RNA was extracted from liver tissues using Trizol reagent (Aidlab, RN0102, Aidlab Biotechnologies Co., Ltd., Beijing, China). Tissues were ground in liquid nitrogen prior to RNA extraction. RNA concentration and purity were measured by absorbance at 260 and 280 nm. cDNA was synthesized using a SuperScript III RT reverse transcription kit (EXONGEN, A502, Chengdu Rongwei Gene Biotechnology Co., Ltd., Chengdu, China). Real-time quantitative PCR was performed on a fluorescence quantitative PCR instrument (Longgene, Q2000B, Hangzhou LongGene Scientific Instruments Co., Ltd., Hangzhou, China), with cDNA as a template and Sybr qPCR mix (ABI-Invitrogen, 4472920, Applied Biosystems, Carlsbad, CA, USA) for amplification [26]. Actin served as the internal control, and primer sequences of target genes are listed in Table 1.

### 2.9. Untargeted Metabolomic Analysis

Liver samples stored at −80 °C were thawed at 4 °C. Samples were vortexed with pre-chilled methanol/acetonitrile/water solution (2:2:1, *v*/*v*), sonicated at low temperature for 30 min, incubated at −20 °C for 10 min, and centrifuged at 14,000× *g* at 4 °C for 20 min, and the supernatant was vacuum-dried. The residue was reconstituted in 100 μL acetonitrile/water (1:1, *v*/*v*), vortexed, and centrifuged at 14,000× *g* at 4 °C for 15 min, and the supernatant was analyzed by liquid chromatography-tandem mass spectrometry (LC-MS/MS) [25]. LC analysis was performed using a Vanquish UHPLC system (Thermo Fisher Scientific, Germering, Germany) equipped with an ACQUITY UPLC BEH Amide column (1.7 μm, 2.1 mm × 100 mm, Waters, Milford, MA, USA). Chromatographic conditions were as follows: column temperature, 25 °C; flow rate, 0.3 mL/min; injection volume, 2 μL. Mobile phase A consisted of water containing 25 mM ammonium acetate and 25 mM ammonia water; mobile phase B was acetonitrile. The gradient elution program was: 0–1.5 min, 98% B; 1.5–12 min, B linearly decreased from 98% to 2%; 12–14 min, 2% B; 14–14.1 min, B linearly increased from 2% to 98%; and 14.1–17 min, 98% B. During analysis, samples were maintained at 4 °C in the autosampler. Mass spectrometry analysis was performed using a Q-Exactive series mass spectrometer (Thermo Fisher Scientific) with positive and negative electrospray ionization (ESI) modes. The ESI parameters were as follows: gas 1 and gas 2 flow rates, 60 units; curtain gas (CUR), 30 psi; ion source temperature, 600 °C; spray voltage (ISVF), ±5500 V (positive and negative modes). The primary mass-to-charge ratio (*m*/*z*) detection range was 80–1200 Da with a resolution of 60,000 and a cumulative scanning time of 100 ms. Secondary mass spectra were acquired using segmented acquisition methods (scanning range 70–1200 Da, resolution 30,000, cumulative scanning time 50 ms, dynamic exclusion time 4 s). Principal component analysis (PCA) and differential metabolite analysis were performed on the LC-MS/MS dataset, and Spearman correlation analysis was conducted to explore associations between gut microbiota and liver metabolites. Metabolites were identified using the Kyoto Encyclopedia of Genes and Genomes (KEGG) database. Criteria for identifying differential metabolites included a high confidence level (VIP > 1) and significant differences in metabolite intensities (*p* < 0.05) [27].

### 2.10. Statistical Analysis

All experiments were repeated three times, and data are presented as mean ± standard deviation (SD). Statistical analyses were performed using IBM SPSS Statistics 27.0.1, GraphPad Prism 8.0.2, and Origin 2024. Univariate ANOVA followed by Tukey’s post hoc test was used to determine statistical significance. Differences were considered statistically significant at *p* < 0.05.

## 3. Results and Discussion

### 3.1. Identification of Chemical Compounds in the Sanhua Tea

The composition of chemical compounds in Sanhua tea is closely related to its physiological bioactivities. A total of 3214 metabolites were identified in Sanhua tea using the UPLC-ESI-Q TRAP-MS/MS platform. These metabolites predominantly included flavonoids (41.14%), alkaloids (14.12%), nucleotides and derivatives (8.06%), amino acids and derivatives (7.85%), phenolic acids (7.19%), and other metabolites. Table 2 summarizes the molecular formulas, masses, and MS/MS fragment ions for selected metabolites. Based on the mean peak areas obtained from three replicate samples, 2-amino-5-[(C-hydroxycarbonimidoyl)amino]pentanimidic acid, L-phenylalaninol, brevifolin carboxylic acid, and cryptochlorogenic acid showed relatively high mass spectrometric responses among the detected compounds. Of these, 2-amino-5-[(C-hydroxycarbonimidoyl)amino]pentanimidic acid was identified as the most abundant metabolite in Sanhua tea. These findings suggest that the lipid-lowering effects of Sanhua tea may be attributed to its flavonoid and alkaloid components. Acacetin, diosmetin, and rutin from the flowers of *Citrus aurantium* L. var. *amara* Engl. have been shown to reduce TC, TG, and LDL-C levels in hyperlipidemic mice [28]. Similarly, aporphine alkaloids from lotus leaves, particularly nuciferine, have been reported to inhibit lipogenesis by activating the AMPK signaling pathway [29]. This evidence supports our hypothesis that tea exerts comparable efficacy in alleviating obesity symptoms.

### 3.2. Effects of Sanhua Tea on Body Weight and Fat in Obese Mice

**Figure 1:**
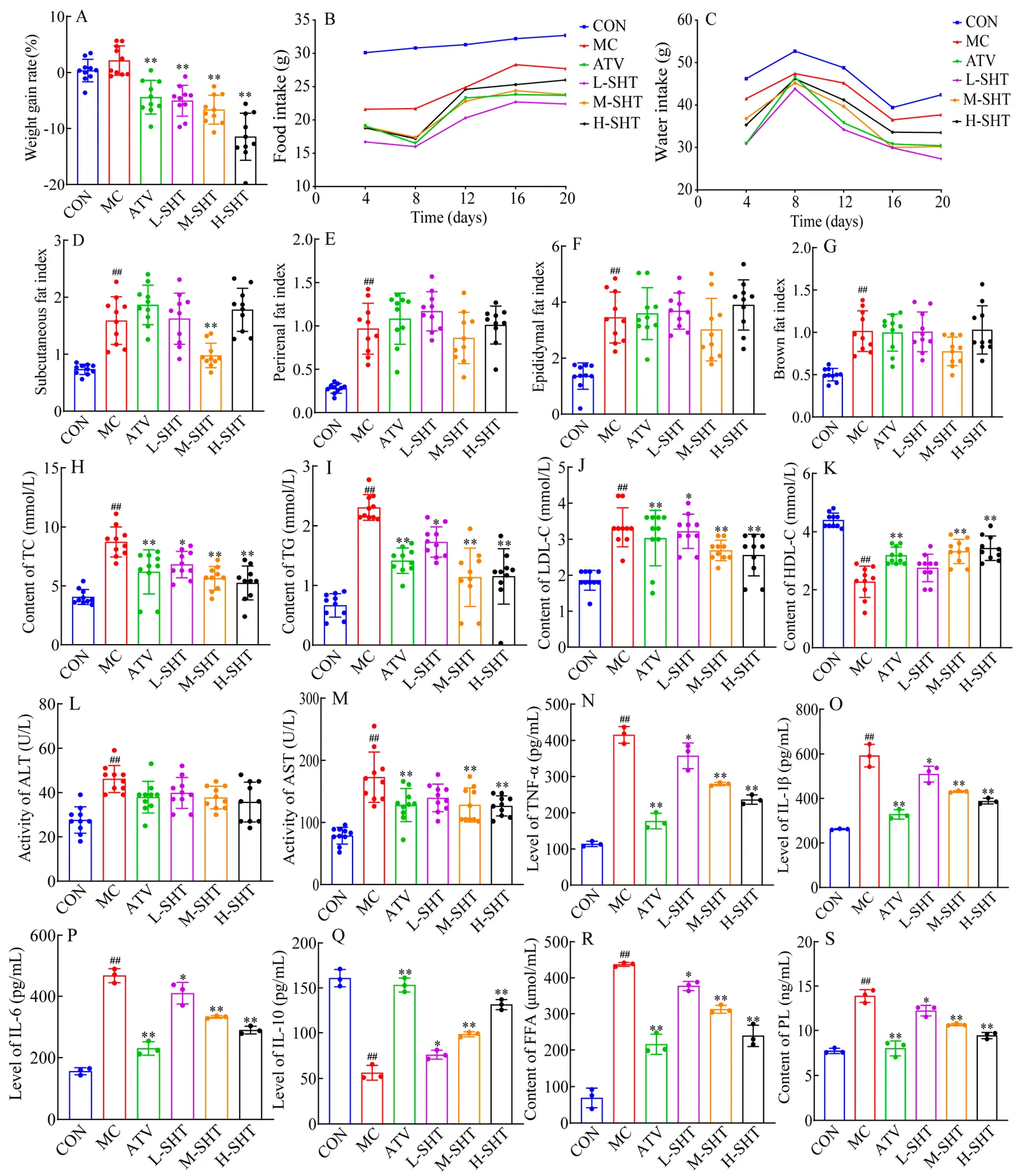
Effects of Sanhua tea on body weight, fat and serum components in obese mice. (**A**) Weight gain rate (*n* = 10); (**B**) food intake for each group during the experiment; (**C**) water intake for each group during the experiment; (**D**) subcutaneous fat index (*n* = 10); (**E**) perirenal fat index (*n* = 10); (**F**) epididymal fat index (*n* = 10); (**G**) brown fat index (*n* = 10); (**H**) TC levels (*n* = 10); (**I**) TG levels (*n* = 10); (**J**) LDL-C levels (*n* = 10); (**K**) HDL-C levels (*n* = 10); (**L**) ALT activity (*n* = 10); (**M**) AST activity (*n* = 10); (**N**) TNF-α levels (*n* = 3); (**O**) IL-1β levels (*n* = 3); (**P**) IL-6 levels (*n* = 3); (**Q**) IL-10 levels (*n* = 3); (**R**) FFA levels (*n* = 3); (**S**) PL levels (*n* = 3). Data are presented as mean ± SD. ## *p* < 0.01 vs. CON group; * *p* < 0.05, ** *p* < 0.01 vs. MC group.

The effects of Sanhua tea on mouse body weight were assessed by calculating the weight gain rate (Figure 1A). The weight gain rate of the MC group was 6.24-fold higher than that of the CON group. After oral administration of ATV or low-, medium-, and high-dose Sanhua tea for 3 weeks, the weight gain rates were significantly reduced (*p* < 0.01). Notably, the H-SHT group showed a more pronounced effect, reducing weight gain to 2.59-fold compared to ATV (*p* < 0.01). This indicates that Sanhua tea administration effectively inhibited weight gain in obese mice, with greater efficacy observed at higher doses. Naringin, which was identified in Sanhua tea, has previously been shown to alleviate diet-induced obesity by stimulating thermogenesis in brown adipose tissue, promoting the browning of white adipose tissue, and modulating the gut microbiota and fecal metabolite profiles [30]. Accordingly, the weight-loss effects of Sanhua tea may involve enhanced thermogenesis and adipose tissue browning, improved lipid metabolism, and modulation of the gut microbial environment. Food and water intake in each group were continuously monitored throughout the treatment period (Figure 1B,C). Overall, both food and water intake remained highest in the CON group, followed by the MC group. Food intake generally increased over time in all groups, with a more pronounced recovery observed in the MC and H-SHT groups after day 12. Water intake followed a broadly similar temporal pattern across groups, initially increasing and subsequently decreasing. Cumulative food intake, cumulative water intake, and food efficiency ratios are presented in Supplementary Table S1.

Compared to the MC group, the subcutaneous fat index of the M-SHT group decreased by 38.51% (*p* < 0.01, Figure 1D). Moreover, M-SHT significantly reduced perirenal, epididymal, and brown adipose tissue indices in mice (Figure 1E–G). These results suggest that different doses of Sanhua tea influence adipose tissue indices to varying extents, with the medium dose demonstrating the most pronounced effects, particularly on subcutaneous adipose tissue accumulation. Consistent with these observations, previous research has shown that naringin significantly reduces epididymal white adipose tissue accumulation in mice fed a high-fat diet by upregulating factors such as UCP1 and PGC-1α. Naringin also enhances thermogenic activity in brown adipose tissue and promotes the browning of inguinal white adipose tissue, effects consistent with those observed following Sanhua tea treatment [30].

### 3.3. Effects of Sanhua Tea on Serum Components in Obese Mice

The serum components of mice were evaluated as shown in Figure 1. Compared with the CON group, serum TC, TG, and LDL-C concentrations significantly increased, while HDL-C levels significantly decreased in obese mice (*p* < 0.01). Sanhua tea treatment effectively improved these lipid abnormalities. Compared with the MC group, the H-SHT group exhibited reductions in TC of 39.86% (*p* < 0.01, Figure 1H), TG of 50.02% (*p* < 0.01, Figure 1I), and LDL-C of 23.12% (*p* < 0.01, Figure 1J), and a 1.51-fold increase in HDL-C levels (*p* < 0.01, Figure 1K). Additionally, TG in the M-SHT group decreased by 50.67%, and HDL-C increased by 1.46-fold compared to the MC group (*p* < 0.01, Figure 1I,K). These results indicate that Sanhua tea significantly reduces serum TC, TG, LDL-C levels, and enhances HDL-C concentrations, with H-SHT showing superior efficacy. Further supporting our findings, flavonoid extracts from the blossoms of *Citrus aurantium* L. var. *amara* Engl. were recently reported to significantly reduce serum TG, TC, and LDL-C levels in mice with high-fat-diet-induced obesity, accompanied by modulation of AMPK- and PPARα-related lipid metabolic pathways [31]. Obesity also elevates serum ALT and AST levels, indicators of liver injury. Sanhua tea effectively reduced ALT and AST concentrations (Figure 1L,M). Specifically, AST in the H-SHT group was reduced by 26.74% (*p* < 0.01, Figure 1M), alleviating obesity-related hepatic damage. Comparable findings were reported previously, indicating that tea extracts significantly lowered serum TG, AST, and ALT levels [32].

Obesity significantly induces metabolic inflammation. Excessive fat accumulation triggers adipocytes to release pro-inflammatory cytokines, including TNF-α, IL-1β, and IL-6, initiating systemic inflammation. ELISA analysis (Figure 1N–Q) revealed markedly elevated inflammatory responses in the MC group compared with the CON group. H-SHT treatment notably suppressed the increases in serum TNF-α, IL-1β, and IL-6 (*p* < 0.01). Moreover, H-SHT significantly elevated the anti-inflammatory cytokine IL-10 (*p* < 0.01), indicating potent anti-inflammatory effects. Previous studies have shown that water extracts of various teas significantly reduce IL-6 levels, consistent with our findings [33]. Obesity results in excessive adipocyte breakdown and subsequent elevated FFA release, increasing pancreatic burden and lipase secretion, thereby raising PL levels. Compared with the MC group, FFA and PL concentrations significantly decreased in the H-SHT group (*p* < 0.01, Figure 1R,S). This suggests that H-SHT effectively mitigates obesity-induced elevations in FFA and PL, demonstrating substantial lipid-lowering potential. Previous studies have reported that flavonoid extracts from Sanhua tea competitively inhibit pancreatic lipase, providing further support for our observations [31,34].

### 3.4. Effects of Sanhua Tea on Gut Microbiota in Obese Mice

**Figure 2:**
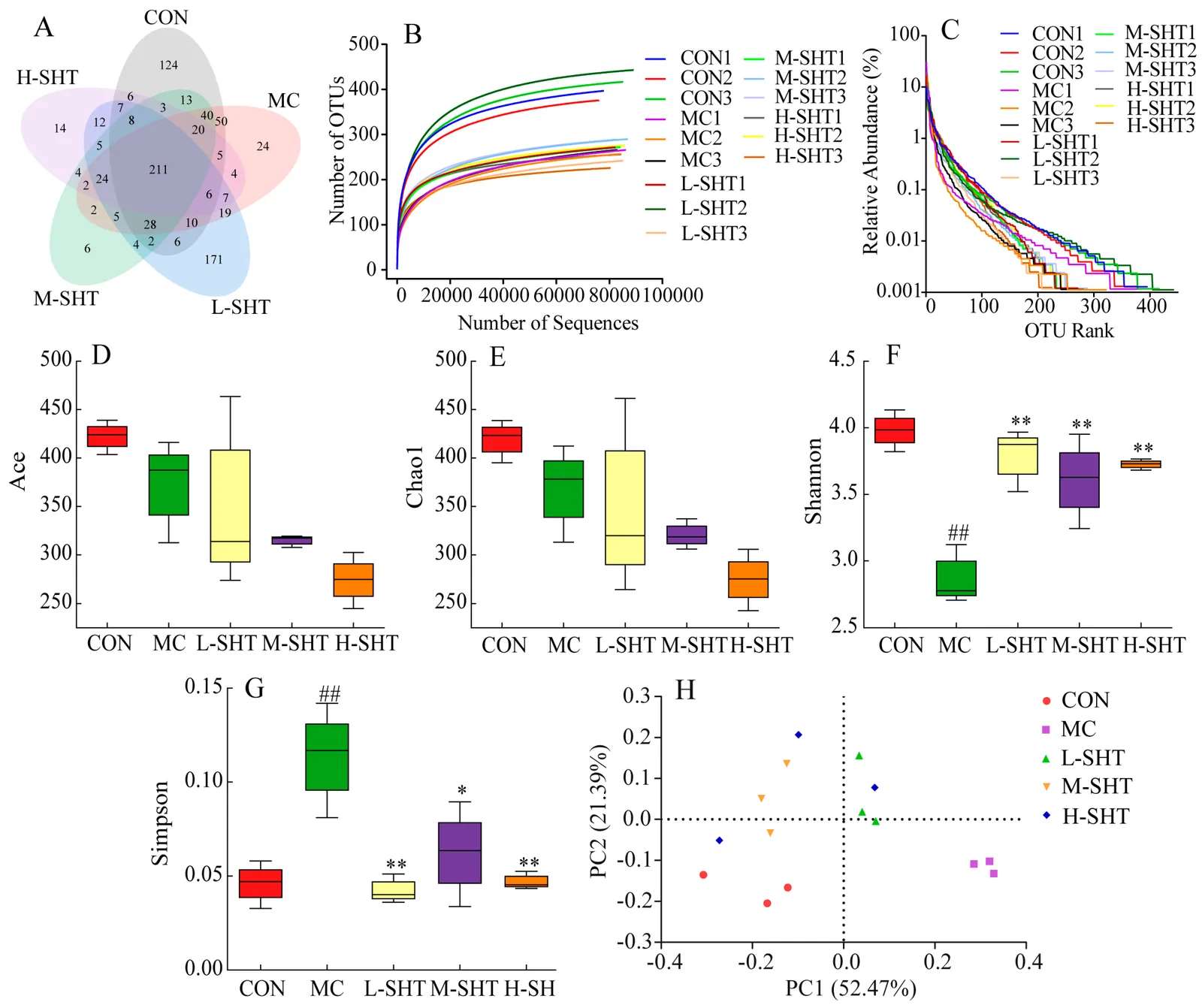
Effects of Sanhua tea on richness and diversity of gut microbiota in obese mice. (**A**,**B**) OTU numbers of gut microbiota; (**C**) OTU rank abundance; (**D**) Ace index; (**E**) Chao1 index; (**F**) Shannon index; (**G**) Simpson index; (**H**) UniFrac distance-based PCoA. Data are presented as mean ± SD (*n* = 3). ## *p* < 0.01 vs. CON group; * *p* < 0.05, ** *p* < 0.01 vs. MC group.

To investigate the modulatory effect of Sanhua tea on gut microbiota, fecal samples from the CON, MC, L-SHT, M-SHT, and H-SHT groups were analyzed by 16S rRNA sequencing. A total of 2236 OTUs at 97% similarity were identified from 15 samples. The Venn diagram (Figure 2A) depicts 211 shared OTUs among all samples.

The rarefaction curves (Figure 2B) gradually plateaued, indicating adequate sequencing depth and sample size for reflecting community structure. Rank–Abundance curves revealed reduced richness and evenness in the MC group compared with the CON group, indicating disruption of gut microbiota structure by a high-fat diet (Figure 2C). The Ace and Chao1 indices showed decreased species richness in the MC group relative to the CON group, a trend alleviated by L-SHT treatment (Figure 2D,E). The Shannon index significantly decreased and the Simpson index significantly increased in the MC group (*p* < 0.01), whereas Sanhua tea reversed these adverse effects, with the most pronounced improvement observed in the L-SHT group (*p* < 0.01). Sanhua tea treatment increased microbial species richness and effectively enhanced microbiota diversity (Figure 2F,G). PCoA also demonstrated that a high-fat-diet-altered normal gut microbiota structure, whereas Sanhua tea administration gradually restored microbiota towards a healthy composition (Figure 2H).

**Figure 3:**
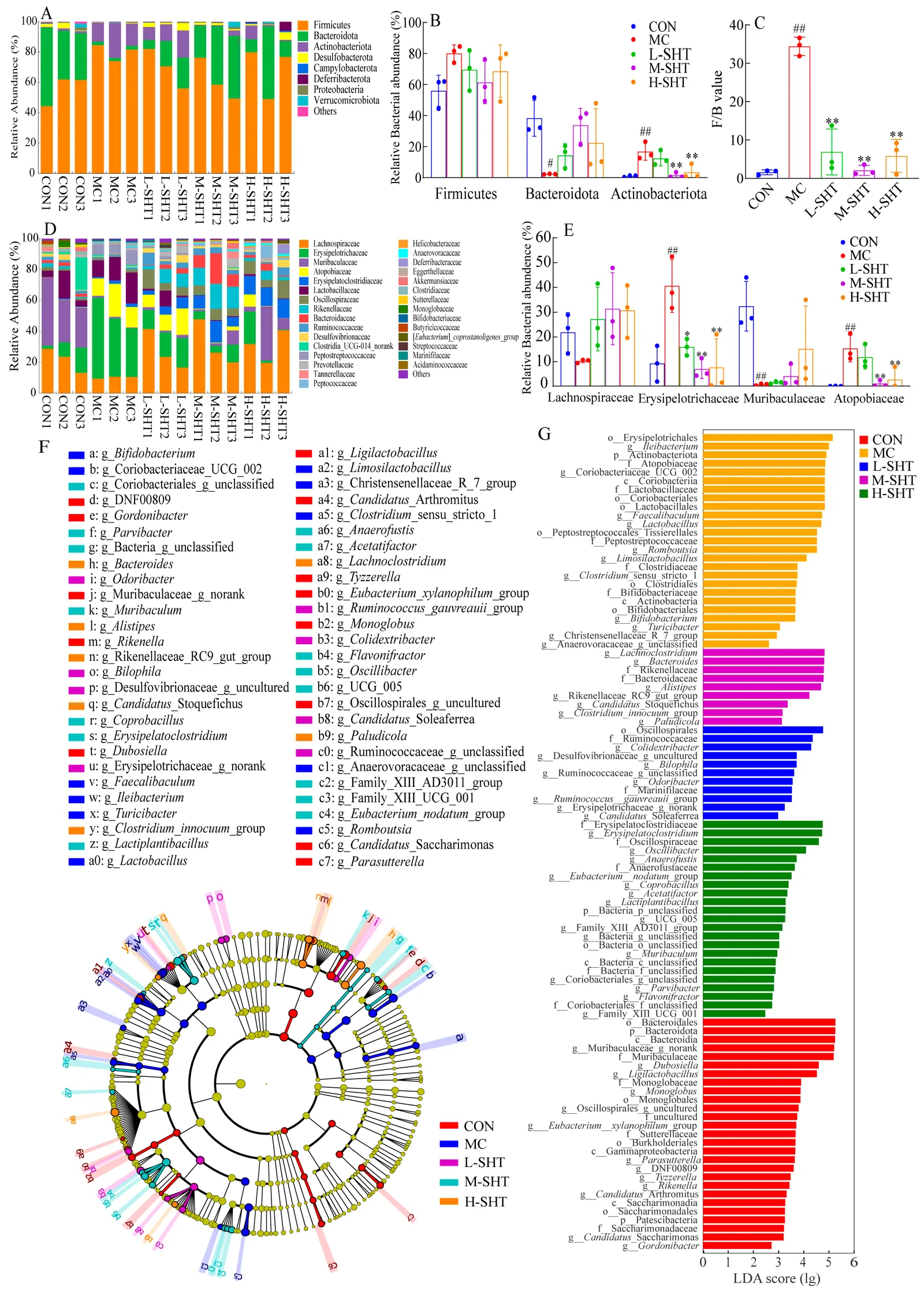
Effects of Sanhua tea on gut microbiota composition. (**A**) Relative abundance at phylum level; (**B**) relative abundance of Firmicutes, Bacteroidota, and Actinobacteriota; (**C**) F/B ratio; (**D**) relative abundance at family level; (**E**) relative abundance of Lachnospiraceae, Erysipelotrichaceae, Muribaculaceae, and Atopobiaceae; (**F**) LEfSe cladogram; (**G**) LDA score histogram (LDA scores > 2). Data are presented as mean ± SD (*n* = 3). # *p* < 0.05, ## *p* < 0.01 vs. CON group; * *p* < 0.05, ** *p* < 0.01 vs. MC group.

To further characterize gut microbiota composition across groups, bacterial taxonomy was analyzed at the phylum and family levels (Figure 3A–E). As shown in Figure 3A, the most abundant phyla were Firmicutes, Bacteroidota, and Actinobacteriota. Firmicutes are primarily involved in food digestion and absorption, fermenting polysaccharides and cellulose to produce heat and fatty acids [35]. Bacteroidota primarily degrade plant-derived dietary components and utilize host-indigestible glycans for energy [36]. The Firmicutes/Bacteroidota (F/B) ratio typically reflects obesity severity. A high-fat diet increased Firmicutes abundance (Figure 3B), a trend mitigated by Sanhua tea treatment, though without significant differences. Compared with the CON group, Bacteroidota and Actinobacteriota abundances significantly differed in the MC group (*p* < 0.05), whereas medium and high doses of Sanhua tea effectively restored gut microbiota structure. The MC group exhibited a significantly elevated F/B ratio compared with the CON group (*p* < 0.01), consistent with previous findings [37,38]. Sanhua tea administration significantly reduced the F/B ratio (*p* < 0.01), effectively alleviating obesity (Figure 3C). At the family level, the most abundant families were Lachnospiraceae, Erysipelotrichaceae, Muribaculaceae, and Atopobiaceae (Figure 3D,E). Lachnospiraceae and Muribaculaceae are beneficial intestinal bacteria; the former participates in carbohydrate metabolism, and increased abundance of the latter alleviates obesity [39]. Conversely, Erysipelotrichaceae and Atopobiaceae negatively impact health. Erysipelotrichaceae are associated with metabolic disorders and cholesterol metabolism, and higher abundance correlates with obesity [40]. Atopobiaceae, a conditional pathogen, is prevalent in obese mice [41]. Compared with the CON group, Lachnospiraceae and Muribaculaceae abundances decreased in the MC group, a trend reversed by Sanhua tea treatment. In contrast, the abundances of Erysipelotrichaceae and Atopobiaceae significantly increased in the MC group, and medium and high doses of Sanhua tea markedly reversed this effect (*p* < 0.01). These findings strongly suggest that Sanhua tea possesses lipid-lowering properties.

LEfSe analysis was applied to identify significantly different microbial taxa among the groups. The microbiota and taxonomic hierarchies with significant differences are shown in Figure 3F,G. A total of 95 significant taxa were shared among all groups (LDA scores > 2, Figure 3G), with 27, 25, 11, 9, and 23 unique taxa identified in the CON, MC, L-SHT, M-SHT, and H-SHT groups, respectively. Substantial differences in microbiota abundance were observed among the groups. For example, the dominant taxa in the MC group included Erysipelotrichales, *Ileibacterium*, Actinobacteriota, and Atopobiaceae, whereas those in the H-SHT group included Erysipelatoclostridiaceae, *Erysipelatoclostridium*, Oscillospiraceae, and *Oscillibacter*. The gut microbiota of obese mice induced by a high-fat diet predominantly comprised Atopobiaceae, Lachnospiraceae, Lactobacillaceae, Ruminococcaceae, and Erysipelotrichaceae, aligning with our observations in the MC group [41].

### 3.5. Effects of Sanhua Tea on Liver Function in Obese Mice

**Figure 4:**
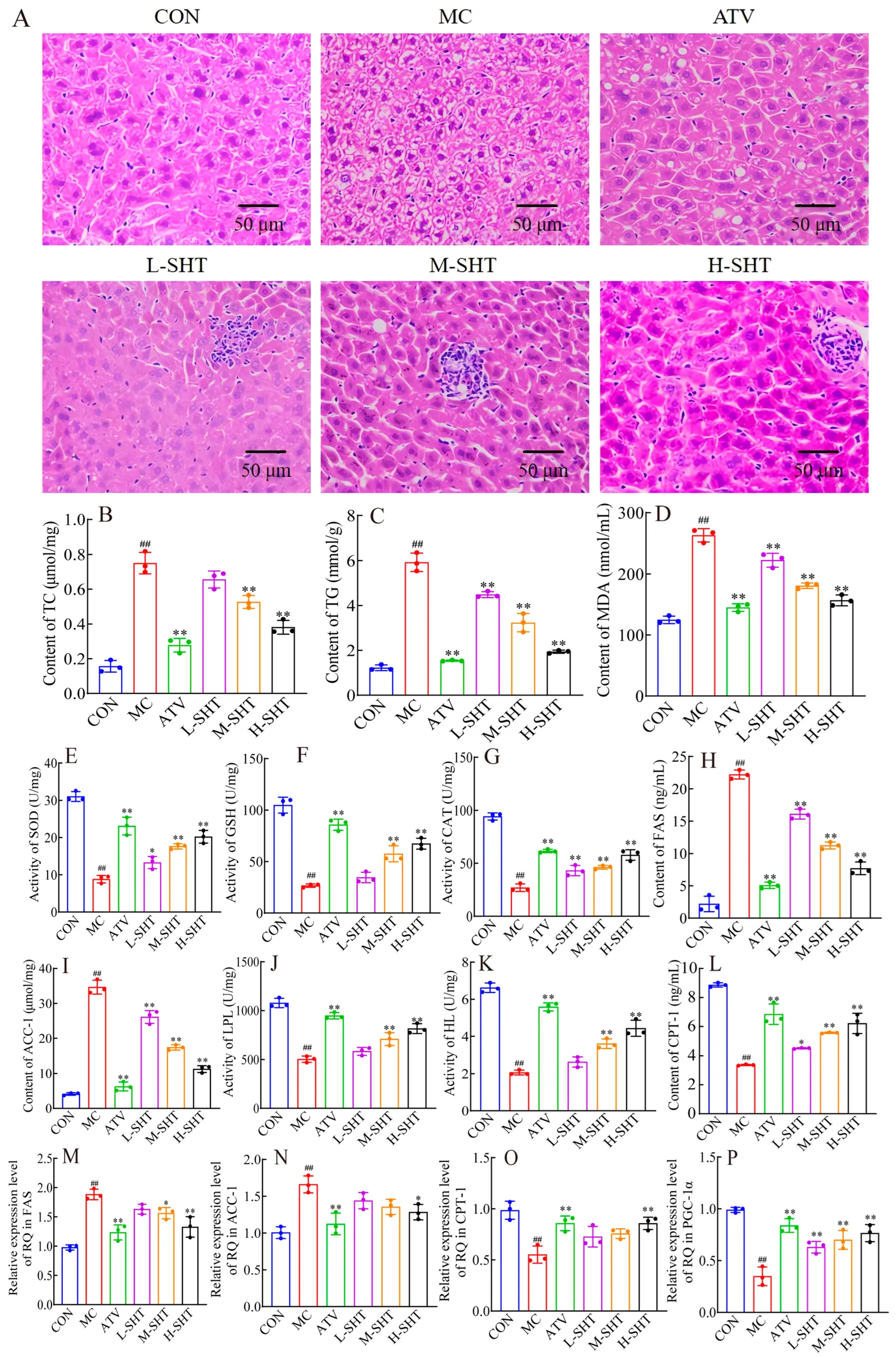
Effects of Sanhua tea on liver function in obese mice. (**A**) Histological sections of liver tissues (magnification ×400); (**B**) TC content; (**C**) TG content; (**D**) MDA content; (**E**) SOD activity; (**F**) GSH activity; (**G**) CAT activity; (**H**) FAS content; (**I**) ACC-1 content; (**J**) LPL activity; (**K**) HL activity; (**L**) CPT-1 content; (**M**) relative mRNA expression of FAS; (**N**) relative mRNA expression of ACC-1; (**O**) relative mRNA expression of CPT-1; (**P**) relative mRNA expression of PGC-1α. Data are presented as mean ± SD (*n* = 3). ## *p* < 0.01 vs. CON group; * *p* < 0.05, ** *p* < 0.01 vs. MC group.

Histopathological features of mouse liver tissues are presented in Figure 4A. Histological examination showed that the CON group maintained intact hepatic architecture, with hepatocytes arranged in regular cords and clearly defined hepatic sinusoids. Hepatocytes exhibited normal morphology, with no apparent steatosis, inflammatory cell infiltration, hepatocellular ballooning, or necrosis. In contrast, the MC group exhibited substantial disruption of hepatic architecture, including hepatocellular swelling, variation in cell size, disorganization of the hepatic cords, and congestion and compression of the hepatic sinusoids. Some hepatocytes showed pale, rarefied cytoplasm, ballooning degeneration, and karyolysis, indicating pronounced hepatocellular injury. Hepatic architecture was partially restored in the ATV group, although mild hepatocellular vacuolation consistent with steatosis persisted. In the L-SHT group, widened and disorganized hepatic cords, compressed hepatic sinusoids, pale cytoplasmic staining, and poorly defined cellular boundaries remained evident. By contrast, the M-SHT and H-SHT groups showed substantially less hepatocellular vacuolation and steatosis, better organized hepatic cords, and relatively well-preserved hepatic morphology. Collectively, these histopathological findings indicate that Sanhua tea, particularly at medium and high doses, attenuated hepatic injury induced by a high-fat diet, consistent with previous findings [32].

The liver, as a primary metabolic organ, directly reflects metabolic abnormalities associated with obesity. Therefore, we further investigated the impact of Sanhua tea on liver function. Compared with the CON group, liver TC and TG contents significantly increased in the MC group, reflecting pronounced obesity (*p* < 0.01). Sanhua tea treatment effectively reduced these lipid accumulations, with M-SHT and H-SHT showing significant effects, and the high-dose group exhibiting the strongest reduction (*p* < 0.01, Figure 4B,C). Previous findings have similarly demonstrated that tea extracts effectively inhibit liver TG accumulation induced by chronic alcohol consumption [32], which aligns with our results.

Obesity is associated with oxidative stress. Increased inflammation in adipose tissue due to obesity promotes free radical production, thereby elevating oxidative stress. MDA, a lipid peroxidation by-product, indicates oxidative liver damage. Compared with the CON group, liver MDA levels significantly increased in the MC group (*p* < 0.01), suggesting obesity-induced oxidative damage. Sanhua tea significantly reduced MDA levels, particularly in the H-SHT group (*p* < 0.01, Figure 4D). SOD, GSH, and CAT neutralize harmful free radicals and exhibit antioxidant properties. Their activities indicate the liver’s antioxidant capacity. Compared with the CON group, SOD, GSH, and CAT activities significantly decreased in the MC group (*p* < 0.01). M-SHT and H-SHT significantly restored these enzyme activities (*p* < 0.01, Figure 4E–G), indicating alleviation of oxidative stress. Previous studies have shown that an ethanol extract of wild rose attenuates oxidative liver injury [42]. Similarly, a jasmine flower decoction significantly decreased hepatic MDA levels while restoring GSH levels and SOD activity [43], consistent with the findings of the present study.

Various hepatic enzymes play crucial roles in metabolic processes. Among these, FAS and ACC-1 regulate fatty acid synthesis, while LPL and HL participate in fatty acid degradation. CPT-1 promotes lipid oxidation and consumption. ELISA analysis (Figure 4H–L) indicated that liver FAS and ACC-1 levels significantly increased in the MC group compared with the CON group (*p* < 0.01), reflecting enhanced fatty acid synthesis. Sanhua tea administration significantly reduced these enzyme levels (*p* < 0.01), with the strongest effects observed at high doses (Figure 4H,I). The activities of LPL and HL significantly decreased in obese mice (*p* < 0.01), indicating impaired fatty acid degradation. However, M-SHT and H-SHT markedly restored the enzymatic activities of LPL and HL (*p* < 0.01, Figure 4J,K). Furthermore, hepatic CPT-1 content significantly decreased in obese mice (*p* < 0.01), indicating reduced lipid utilization, whereas H-SHT significantly elevated CPT-1 levels (*p* < 0.01, Figure 4L). Taurine reportedly reduces fat deposition induced by a high-fat diet via decreasing FAS levels [44]. Rose petal extract has also been reported to suppress lipid synthesis by downregulating FAS expression and increasing ACC phosphorylation, while simultaneously promoting AMPK activation and CPT1A expression, in agreement with our findings [45].

To further elucidate the mechanism underlying the beneficial effects of Sanhua tea on obesity-related metabolic abnormalities, hepatic metabolism-related genes were analyzed by real-time PCR. Compared with the CON group, hepatic FAS and ACC-1 mRNA expression significantly increased in the MC group (*p* < 0.01), accelerating lipid synthesis and accumulation. H-SHT treatment significantly inhibited these increases in FAS and ACC-1 expression (*p* < 0.01, Figure 4M; *p* < 0.05, Figure 4N). Previous research demonstrated that *Pachyrhizus erosus* prevents obesity by suppressing FAS expression [46]. Similarly, cinnamic acid reduces FAS and ACC-1 expression, consistent with our observations [41]. High-fat diets significantly reduced hepatic CPT-1 and PGC-1α mRNA expression (*p* < 0.01, Figure 4O,P), supporting previous findings [47]. Sanhua tea administration significantly elevated PGC-1α expression (*p* < 0.01, Figure 4P) and substantially increased CPT-1 expression in the high-dose group (*p* < 0.01, Figure 4O), thus promoting lipid metabolism. Studies showed that milk fat globule membrane ameliorates adipose tissue dysfunction caused by a high-fat diet through upregulating PGC-1α [48], enhancing thermogenesis and energy expenditure [49], paralleling Sanhua tea’s lipid-lowering mechanism.

### 3.6. Effects of Sanhua Tea on Liver Metabolites in Obese Mice

**Figure 5:**
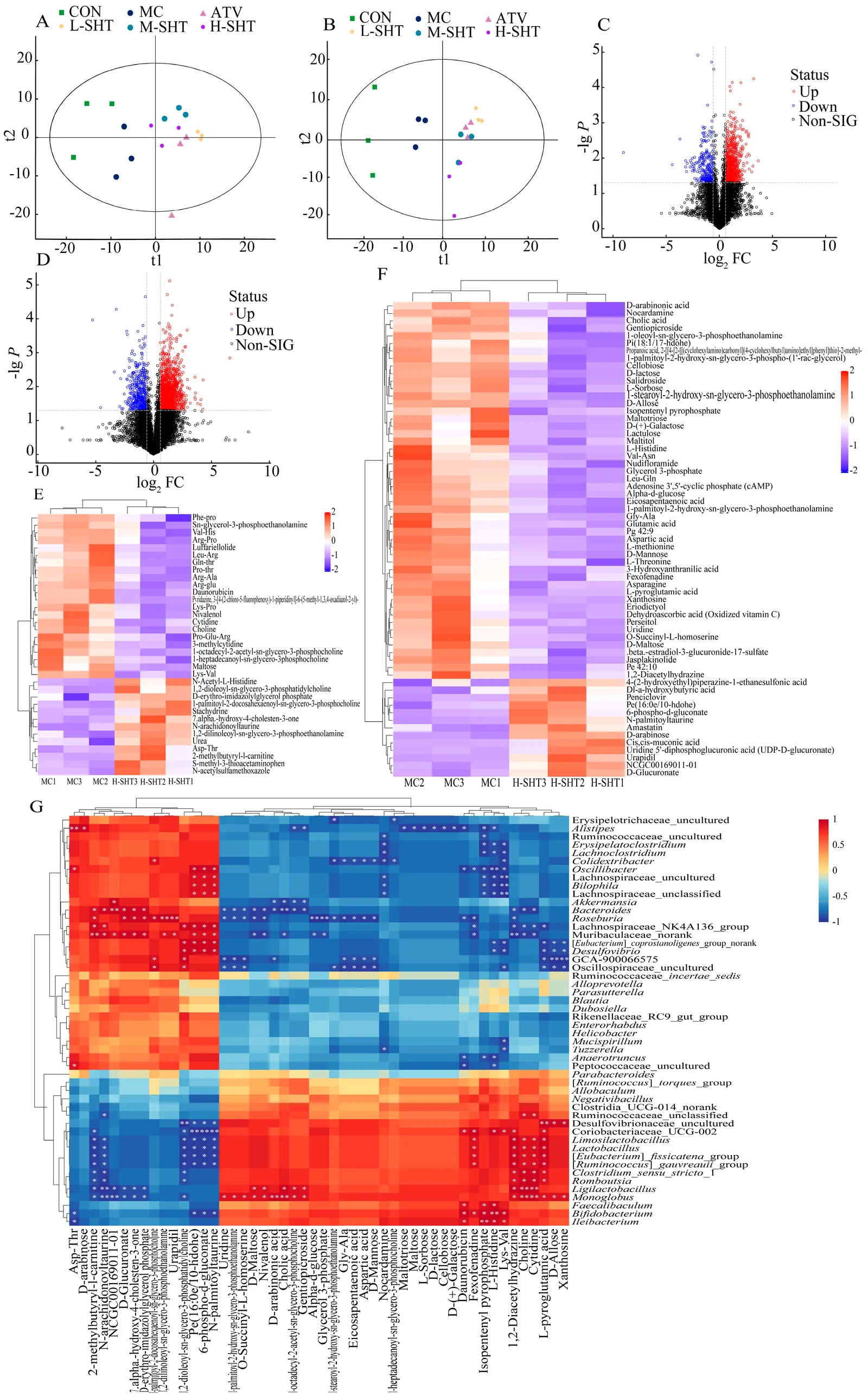
Effects of Sanhua tea on the hepatic metabolomic profile in obese mice. (**A**) PCA score plot in positive ion mode; (**B**) PCA score plot in negative ion mode; (**C**) volcano plot of differential metabolites (H-SHT vs. MC) in positive ion mode; (**D**) volcano plot of differential metabolites (H-SHT vs. MC) in negative ion mode; (**E**) heatmap illustrating 35 significantly altered metabolites (H-SHT vs. MC) in positive ion mode; (**F**) heatmap illustrating 63 significantly altered metabolites (H-SHT vs. MC) in negative ion mode; (**G**) Spearman correlation analysis between gut microbiota and liver metabolites. Data are presented from three mice per group; * *p* < 0.05, ** *p* < 0.01.

To further explore effects of Sanhua tea on the hepatic metabolomic profile in obese mice, an untargeted metabolomics analysis using LC-MS/MS was performed. PCA results showed distinct clustering among the six groups in both positive and negative ion modes, indicating robust sample stability and repeatability. Additionally, significant differences in metabolite profiles were observed among the groups (Figure 5A,B).

A total of 2119 metabolites were identified, comprising 1172 in positive ion mode and 947 in negative ion mode. Predominant metabolite classes included lipids and lipid-like molecules (24.45%) and organic acids and derivatives (23.93%). Using fold-change (FC) > 1.5 and *p* < 0.05 criteria, significantly upregulated metabolites were identified. Compared with the MC group, the H-SHT group exhibited increases in 130 metabolites (positive ion mode) and 127 metabolites (negative ion mode). Similarly, based on FC < 0.67 and *p* < 0.05, 29 metabolites (positive ion mode) and 33 metabolites (negative ion mode) were significantly downregulated in the H-SHT group relative to the MC group (Figure 5C,D). In addition, differential metabolites were screened using OPLS-DA VIP > 1 and *p* < 0.05. Among the top 35 significantly altered metabolites in positive ion mode (log_2_ FC), H-SHT significantly increased the levels of stachydrine and 7α-hydroxy-4-cholesten-3-one, while markedly reducing Lys-Val, Arg-Glu, maltose, and Lys-Pro. In negative ion mode, among the top 63 significantly altered metabolites, H-SHT significantly elevated the content of uridine 5′-diphosphoglucuronic acid (UDP-D-glucuronate) and substantially decreased D-lactose levels (Figure 5E,F).

### 3.7. Correlation Analysis Between Gut Microbiota and Liver Metabolites in Obese Mice

Spearman correlation analysis was performed to evaluate associations between the top 50 gut microbial genera and liver metabolites in the MC and H-SHT groups (Figure 5G). At the genus level, metabolites such as Asp-Thr, NCGC00169011-01, 1,2-dilinoleoyl-sn-glycero-3-phosphoethanolamine, urapidil, and 2-methylbutyryl-l-carnitine positively correlated with beneficial gut microbiota, including *Alistipes*, *Bacteroides*, *Roseburia*, and Muribaculaceae_norank. Additionally, choline, cytidine, L-histidine, and isopentenyl pyrophosphate positively correlated with beneficial microbiota such as *Ligilactobacillus*, *Bifidobacterium*, and *Ileibacterium*. Conversely, Lys-Val, D-allose, and xanthosine negatively correlated with harmful gut microbiota (*Bilophila* and GCA-900066575), while 1,2-dioleoyl-sn-glycero-3-phosphatidylcholine, Pe(16:0e/10-hdohe), 6-phospho-D-gluconate, and N-palmitoyltaurine negatively correlated with harmful microbiota such as Desulfovibrionaceae_uncultured and Coriobacteriaceae_UCG-002. Aspartic acid (Asp) is a non-essential amino acid critical for protein synthesis, energy metabolism, and liver protection [50,51,52]. Threonine (Thr) modulates immune function, hepatic lipid metabolism, and intestinal health [53]. The beneficial genus *Alistipes* is linked to reduced blood TG [54], whereas *Bacteroides* species regulate body weight, adiposity, and inflammation [55]. Specifically, *Bacteroides stercoris* KGMB02265 exhibits anti-obesity potential by reducing lipid accumulation markers and leptin/TG levels [56]. *Roseburia* represents a promising probiotic candidate for anti-obesity applications. *Roseburia hominis* inhibits white adipose tissue expansion and prevents the whitening of brown adipose tissue, thereby mitigating weight gain, glycolipid metabolic disorders, and fatty liver [57]. Choline, a semi-essential nutrient, plays a crucial role in lipid and carbon metabolism; choline deficiency is associated with liver injury, whereas adequate choline intake reduces obesity risk [58,59]. Cytidine, converted into uridine by cytidine deaminase, alleviates dyslipidemia and hepatic steatosis by modulating gut microbiota composition, particularly by increasing populations that produce short-chain fatty acids [60]. L-Histidine (L-His), an essential amino acid with unique biochemical roles, counteracts cellular inflammation induced by non-esterified fatty acids and alleviates related metabolic disorders by inhibiting Gab2 expression [61]. Species of the genus *Ligilactobacillus* show potential as targeted probiotics for obesity and metabolic disorders. Specifically, *Ligilactobacillus animalis* LA-1 exhibits anti-obesity effects through modulation of gut microbiota composition, enhancement of short-chain fatty acid production, and metabolic reprogramming in the liver [62]. Additionally, *Ligilactobacillus salivarius* LCK11 prevents obesity by promoting secretion of peptide YY (PYY), thereby suppressing food intake and modulating gut microbiota [63]. *Bifidobacterium*, a key gut microbiota genus, reduces serum ghrelin levels and elevates serum leptin concentrations, improving blood lipid profiles and body fat composition, thus serving as a recognized probiotic for obesity management [64]. Increased abundance of beneficial microorganisms such as *Ileibacterium* and Lachnospiraceae UCG-006 has been shown to mitigate high-fat diet-induced obesity [65,66]. Valine (Val), a branched-chain amino acid involved in protein synthesis and energy production, mitigates oxidative stress by significantly reducing mitochondrial reactive oxygen species and 4-hydroxynonenal expression [67]. D-Allose, a rare sugar utilizing glucose transporter proteins, alleviates cardiomyocyte hypertrophy by reducing intracellular glucose flux and glycolysis [68]. Reduced abundance of harmful genera such as *Bilophila* and GCA-900066575 is beneficial in alleviating obesity symptoms [69,70]. Decreased abundance of Desulfovibrionaceae effectively mitigates obesity risk from high-fat diets [71]. Additionally, increased *Lactobacillus* abundance coupled with reduced Coriobacteriaceae_UCG-002 improves gut microbiota imbalance [72]. Therefore, these findings indicate that Sanhua tea alleviates obesity-related symptoms in mice, likely through modulation of host metabolites involved in liver protection, immunity enhancement, anti-inflammation, glucose regulation, oxidative stress reduction, intestinal health maintenance, and anti-tumor activities.

### 3.8. Effects of Sanhua Tea on Metabolic Pathways in Obese Mice

**Figure 6:**
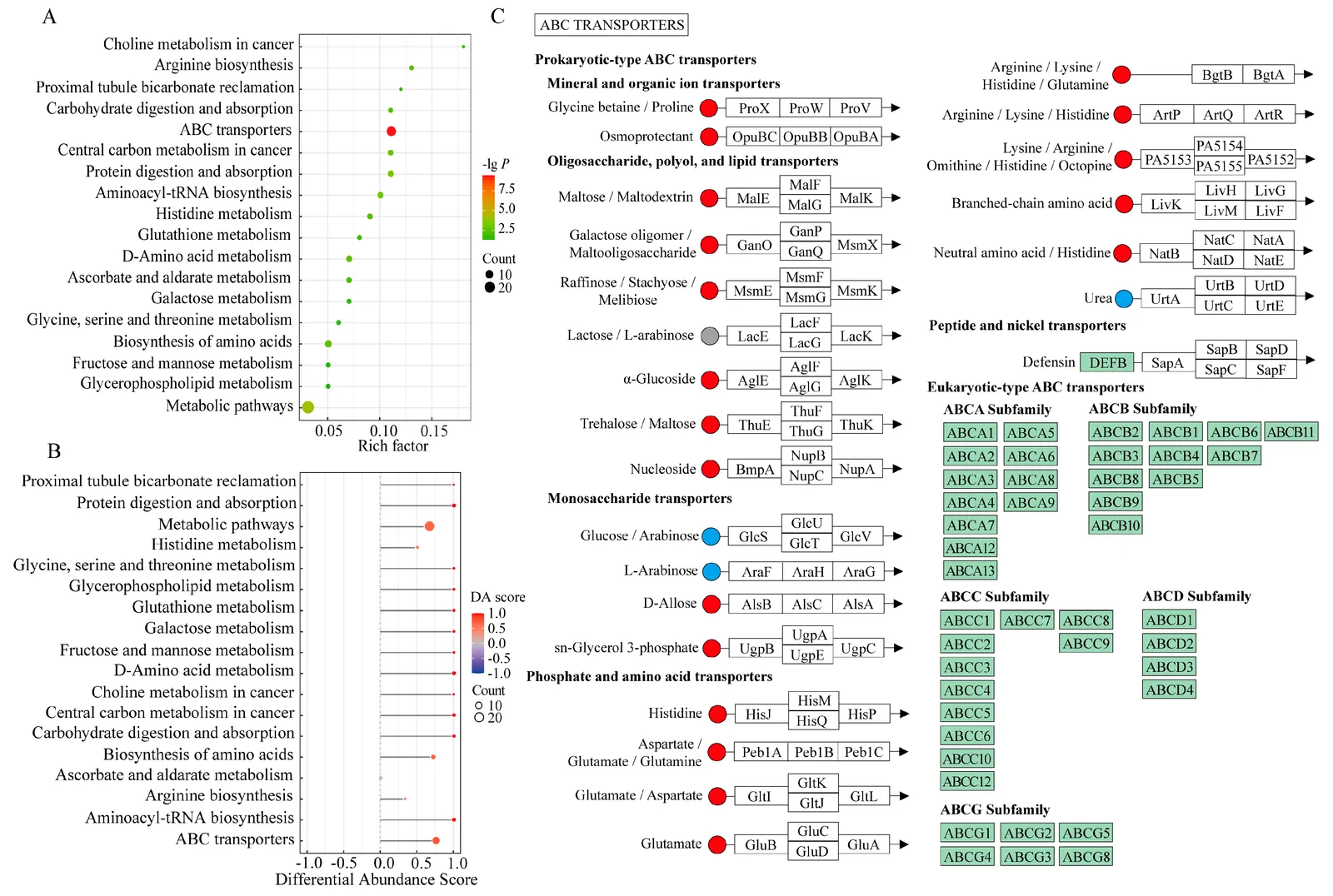
Effects of Sanhua tea on metabolic pathways in obese mice. (**A**) Pathway impact from topology analysis in H-SHT vs. MC; (**B**) DA score plots for H-SHT vs. MC; (**C**) KEGG annotation of differential metabolites in the ABC transporter pathway.

KEGG pathway analysis was performed to further elucidate the functional roles of significantly altered metabolites. Differentially abundant metabolites between the MC and H-SHT groups were enriched in 18 primary pathways (Figure 6A). Among these, ABC transporters, metabolic pathways, and protein digestion and absorption were the most significantly enriched (*p* < 0.01), all of which are closely related to lipid metabolism. Compared with the MC group, the H-SHT group exhibited a substantial increase in the number of up-regulated metabolites within five pathways—protein digestion and absorption, D-amino acid metabolism, central carbon metabolism in cancer, carbohydrate digestion and absorption, and aminoacyl-tRNA biosynthesis—suggesting their potential importance in Sanhua tea-mediated regulation of lipid metabolism disorders (Figure 6B). ABC transporters are predominantly expressed in lipid-processing cells, playing a critical role in lipid transport and metabolic diseases [73]. Dysregulated metabolic pathways are closely associated with metabolic syndrome, which commonly accompanies obesity [74]. Additionally, the gut microbiota may influence obesity susceptibility through the protein digestion and absorption pathway [75]. Regulation of D-amino acid metabolism has been linked to improved lipid metabolic profiles [76]. Central carbon metabolism in cancer pathway enrichment indicates potential alterations in energy metabolism [77]. Restoring disrupted metabolites involved in carbohydrate digestion and absorption to normal levels is related to anti-obesity mechanisms [78]. Moreover, aminoacyl-tRNA biosynthesis has been implicated in serum metabolic alterations induced by a high-fat diet [79]. These pathways may thus represent key mechanisms by which Sanhua tea alleviates obesity in mice fed a high-fat diet.

We next focused on the ABC transporter pathway and its associated metabolites, given its significant enrichment (lowest *p*-value). Specifically, ABCA1 and ABCG1 transporters have been extensively studied in metabolic diseases. ABCA1, a critical regulator of adipocyte differentiation and lipid accumulation, shows decreased expression closely associated with obesity [72], consistent with our findings. KEGG annotations (Figure 6C) of differential metabolites in the ABC transporter pathway revealed that, compared with the MC group, H-SHT regulated the activity of mineral and organic ion transporters by increasing glycine betaine/proline and osmoprotectants, and modulated phosphate and amino acid transporter pathways by decreasing urea content. Glycine betaine, a metabolite derived from glycine, significantly improves glucose and lipid metabolism, reducing obesity [80]. Elevated urea levels are linked to key metabolic syndrome components, including obesity, insulin resistance, dyslipidemia, and hypertension; thus, reduced urea may reflect enhanced protein utilization during anabolic metabolism [81]. Amino acid transporters (AATs), membrane-bound proteins facilitating amino acid transport, are dysregulated in obesity, diabetes, and cancer, altering amino acid metabolism and contributing to disease pathology [82]. Therefore, these metabolite alterations could significantly contribute to the anti-obesity efficacy of Sanhua tea. However, glutamic acid concentration positively correlates with central adiposity [83]. Branched-chain amino acids (BCAAs), consisting of leucine, isoleucine, and valine, typically accumulate in conditions such as insulin resistance, obesity, and type 2 diabetes [84]. In our analysis, glutamate/aspartate and BCAA contents increased, possibly due to multiple factors, including the drug’s mechanism of action, compensatory responses, individual variability, and gut microbiota modulation. These changes represent a dynamic balance maintained by the body under drug intervention, and their exact mechanisms warrant further investigation. Future studies should explore the impact of Sanhua tea on gut-derived metabolites. Additionally, because Sanhua tea was evaluated as a complete herbal formulation in this study, the relative contributions of its individual herbal components and identified constituents to the observed effects could not be determined. Therefore, attributing these effects to any specific herb or compound would be speculative and should be validated in future studies examining individual components and constituents.

## 4. Conclusions

In conclusion, this study demonstrates that Sanhua tea alleviates obesity symptoms induced by an improper diet by improving blood lipid profiles and reducing inflammation. In addition, Sanhua tea regulates gut microbiota homeostasis in obese mice and ameliorates obesity-related liver injury. Untargeted metabolomics analysis further revealed that Sanhua tea significantly modulates metabolites involved in multiple metabolic pathways. These metabolites may underlie the anti-obesity effects of Sanhua tea, with the ABC transporter signaling pathway playing a key role. Therefore, this study provides further evidence supporting the potential application of Sanhua tea in human therapy and offers a reference for its functional evaluation. Several limitations of this study should be acknowledged. First, no a priori sample-size calculation or statistical power analysis was conducted. Although 10 mice per group were included in the principal animal-level assessments, the statistical power to detect small treatment effects remains uncertain. Second, owing to constraints in sample availability and analytical resources, several histological, cytokine, qPCR, microbiome, and metabolomic analyses were performed using only three biological replicates per group. This limited sample size may not adequately capture inter-individual variability and reduces the statistical robustness of these subset analyses. This limitation is particularly relevant to the high-dimensional microbiome and metabolomic datasets, which also involve a substantial multiple-testing burden. Therefore, these findings should be regarded as exploratory evidence that complements the physiological and biochemical results rather than as definitive confirmation of the underlying mechanisms. Future studies should incorporate longer intervention and follow-up periods, prospectively determined sample sizes, and larger biological cohorts to confirm the durability of the observed effects and validate the associated microbial and metabolic changes. Notably, although the food-intake data help assess whether reduced caloric intake contributed to the observed weight loss, they cannot independently distinguish the effects of altered energy intake from those of changes in energy expenditure or metabolic efficiency.

## Figures and Tables

**Table 1:** Primer sequences used for real-time PCR.

Gene Name	Primers	Sequences
*FAS* ^1^	Forward (5′-3′)	ACTAATAGCATCTCCGAGAGT
	Reverse (5′-3′)	GGGCCTCCTTGATATAATCCTT
*ACC-1* ^2^	Forward (5′-3′)	TTGCTGTAGAAACCCGAAC
	Reverse (5′-3′)	CTGCTGGATTATCTTGGCT
*CPT-1* ^3^	Forward (5′-3′)	ACTCCGCTCGCTCATTCCG
	Reverse (5′-3′)	GACTGTGAACTGGAAGGCCA
*PGC-1α* ^4^	Forward (5′-3′)	TTCATCACCTACCGTTACACCT
	Reverse (5′-3′)	CGACCTGCGTAAAGTATATCCAT
actin	Forward (5′-3′)	CTCCTGAGCGCAAGTACTCT
	Reverse (5′-3′)	TACTCCTGCTTGCTGATCCAC

^1^ *FAS*: fatty acid synthetase; ^2^ *ACC-1*: acetyl-CoA carboxylase-1; ^3^ *CPT-1*: carnitine palmitoyltransferase-1; ^4^ *PGC-1α*: peroxisome proliferator-activated receptor γ coactivator-1α.

**Table 2:** Typical constituents identified in Sanhua tea by UPLC-ESI-Q TRAP-MS/MS.

Compound	Molecular Formula	Ion Mode	*m*/*z*	MS/MS Fragment Ions	Relative Peak Area (×10^7^)
2-amino-5-[(C-hydroxycarbonimidoyl)amino]pentanimidic acid	C_6_H_14_N_4_O_2_	[M + H]+	175.12	70.07	21.42
L-Phenylalaninol	C_9_H_13_NO	[M + H]+	152.11	91.06	17.58
Nor-O-methylarmepavine	C_19_H_23_NO_3_	[M + H]+	314.18	107.05	12.90
(S)-N-Methylcoclaurine	C_18_H_21_NO_3_	[M + H]+	300.16	107.05	12.07
Roemerine	C_18_H_17_NO_2_	[M + H]+	280.13	249.09	11.89
Tricetin 3′-glucoside	C_21_H_20_O_12_	[M + H]+	465.1	303.05	11.22
Diosmetin-7-O-galactoside	C_22_H_22_O_11_	[M + H]+	463.12	301.07	10.50
Armepavine	C_19_H_23_NO_3_	[M + H]+	314.17	283.14	10.45
Hesperetin 7-O-glucoside	C_22_H_24_O_11_	[M + H]+	465.14	303.09	9.73
4-[3-(2,3-dihydroxyphenyl)prop-2-enoyloxy]-1,3,5-trihydroxycyclohexane-1-carboxylic acid	C_16_H_18_O_9_	[M + H]+	355.1	163.04	9.68
Brevifolin carboxylic acid	C_13_H_8_O_8_	[M − H]−	291.01	247.02	11.32
Cryptochlorogenic acid (4-O-Caffeoylquinic acid)	C_16_H_18_O_9_	[M − H]−	353.09	191.06	10.21
3,4,5-Trihydroxy-6-[2-hydroxy-5-(3,5,7-trihydroxy-4-oxochromen-2-yl)phenoxy]oxane-2-carboxylic acid	C_21_H1_8_O_13_	[M − H]−	477.07	301.05	7.83
Citric Acid	C_6_H_8_O_7_	[M − H]−	191.02	111.01	7.74
Hesperidin	C_28_H_34_O_15_	[M − H]−	609.18	301.09	7.18
Malate	C_4_H_6_O_5_	[M − H]−	133.01	71.01	6.77
2,5-Dibenzoylglucaric acid	C_20_H_18_O_10_	[M − H]−	417.08	121.03	6.27
Naringin	C_27_H_32_O_14_	[M − H]−	579.17	271.06	6.06
2,3-Dibenzoylglucaric acid	C_20_H_18_O_10_	[M − H]−	417.08	121.03	5.82
Narirutin	C_27_H_32_O_14_	[M − H]−	579.17	271.06	5.53

## Data Availability

The original contributions presented in this study are included in the article/Supplementary Materials. Further inquiries can be directed to the corresponding author.

## Supplementary Materials

The following supporting information can be downloaded at https://www.mdpi.com/article/10.3390/foods15162912/s1, Table S1: Cumulative food intake, water intake, and food efficiency ratios for each group; Figure S1: Contents of TC, TG, and LDL-C in serum of mice at the end of the sixth week. (A) TC levels; (B) TG levels; (C) LDL-C levels. Data are presented as mean ± SD (*n* = 10). ** *p* < 0.01 vs. CON group.

## Abbreviations

The following abbreviations are used in this manuscript:

SHT	Sanhua tea
TC	Total cholesterol
TG	Triglyceride
LDL-C	Low-density lipoprotein cholesterol
HDL-C	High-density lipoprotein cholesterol
TNF-α	Tumor necrosis factor-α
IL-1β	Interleukin-1β
IL-6	Interleukin-6
IL-10	Interleukin-10
MDA	Malondialdehyde
SOD	Superoxide dismutase
GSH	Glutathione
CAT	Catalase
FAS	Fatty acid synthase
PGC-1α	Peroxisome proliferator-activated receptor γ coactivator-1α
CPT-1	Carnitine palmitoyltransferase-1
F/B	Firmicutes/Bacteroidota
Lys-Val	Lysine–valine
GLP-1	Glucagon-like peptide-1
CAD	Collision-activated dissociation
MRM	Multiple reaction monitoring
DP	Declustering potentials
CE	Collision energy
SPF	Specific pathogen-free
CON	Blank control
MC	Model control
ATV	Atorvastatin
L-SHT	Low-dose Sanhua tea
M-SHT	Medium-dose Sanhua tea
H-SHT	High-dose Sanhua tea
ALT	Alanine aminotransferase
AST	Aspartate aminotransferase
FFA	Free fatty acid
PL	Pancreatic lipase
GSH-PX	Glutathione peroxidase
ACC-1	Cetyl-CoA carboxylase-1
LPL	Lipoprotein lipase
HL	Hepatic lipase
PCoA	Principal coordinate analysis
LEfSe	Linear discriminant analysis effect size
H&E	Hematoxylin–eosin
PFA	Paraformaldehyde
PCR	Polymerase chain reaction
LC-MS/MS	Liquid chromatography-tandem mass spectrometry
ESI	Electrospray ionization
CUR	Curtain gas
ISVF	Spray voltage
PCA	Principal component analysis
KEGG	Kyoto Encyclopedia of Genes and Genomes
SD	Standard deviation
FC	Fold-change
UDP-D-glucuronate	Uridine 5′-diphosphoglucuronic acid
Asp	Aspartic acid
Thr	Threonine
L-His	L-Histidine
PYY	Peptide YY
Val	Valine
AATs	Amino acid transporters
BCAAs	Branched-chain amino acids
