# Supplementary material for: Sanhua Tea Alleviates Obesity Symptoms by Improving Blood Lipid Levels, Gut Microbiota, and Liver Metabolome in Mice
Source: Foods. 2026 Aug 20;15(16):2912. doi: 10.3390/foods15162912 (PMC13511786; doi:10.3390/foods15162912)
Supplement: Supplementary file 1 [file foods-15-02912-s001.zip › foods-4453474-supplementary.pdf]

Table S1. Cumulative food intake, water intake, and food efficiency ratios for each group.

Group	Cumulative Food Intake (g)	Cumulative Water Intake (g)	Food Efficiency Ratio (%)
CON	610.6	884.6	0.13
MC	498.7	770.7	1.26
ATV	432.9	646.0	-3.12
L-SHT	397.8	627.6	-3.54
M-SHT	427.9	694.8	-5.61
H-SHT	457.9	761.4	-8.80

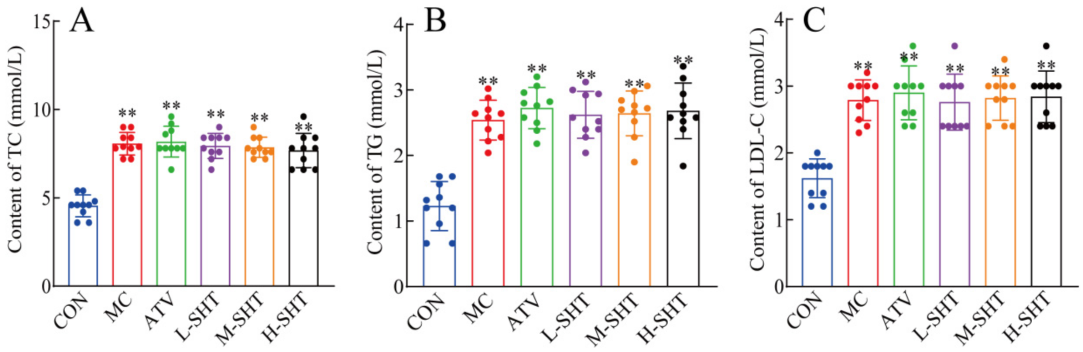

Figure S1. Contents of TC, TG, and LDL-C in serum of mice at the end of the sixth week. (**A**) TC levels; (**B**) TG levels; (**C**) LDL-C levels. Data are presented as mean ± SD (n=10). **$p < 0.01$ vs. CON group.
